# Central memory CD4+ T cells are preferential targets of double infection by HIV-1

**DOI:** 10.1186/s12985-015-0415-0

**Published:** 2015-11-11

**Authors:** Aiman A. Haqqani, Samantha L. Marek, Jagadish Kumar, Miles Davenport, Heng Wang, John C. Tilton

**Affiliations:** Center for Proteomics and Bioinformatics, School of Medicine, Case Western Reserve University, 10900 Euclid Ave, BRB 919, Cleveland, OH 44106 USA; Complex Systems in Biology Group, Center for Vascular Research, University of New South Wales, Sydney, NSW 2052 Australia; DDC Clinic—Center for Special Needs Children, Middlefield, OH 44062 USA

## Abstract

**Background:**

Template switching between two distinct HIV-1 RNA genomes during reverse transcription gives rise to recombinant viruses that greatly expand the genetic diversity of HIV-1 and have adverse implications for drug resistance, immune escape, and vaccine design. Virions with two distinct genomes are produced exclusively from cells infected with two or more viruses, or ‘doubly infected’ cells. Previous studies have revealed higher than expected frequencies of doubly infected cells compared to frequencies based on chance alone, suggesting non-random enhancement of double infection.

**Methods:**

We investigated double infection of unstimulated primary CD4+ T cells using reporter viruses carrying genes for different fluorescent proteins, EGFP and mCherry, combined with sophisticated modeling techniques based on Poisson distribution. Additionally, through the use of multiparameter flow cytometry we examined the susceptibility of naïve and memory subsets of CD4+ T cells to double infection by HIV.

**Results:**

Using our double infection system, we confirm non-random enhancement of multiple infection events. Double infection of CD4+ T cells was not found to be a consequence of suboptimal provirus expression rescued by Tat *in trans*—as has been reported in cell lines—but rather due to a heterogeneous cell population in which only a fraction of primary peripheral blood CD4+ T cells are susceptible to HIV infection regardless of viral titer. Intriguingly, double infection of CD4+ T cells occurred preferentially in memory CD4+ T cells—particularly the central memory (T_CM_) subset—but was not a consequence of SAMHD1-mediated restriction of HIV infection in naïve cells.

**Conclusions:**

These findings reveal that double infection in primary CD4+ T cells is primarily a consequences of cellular heterogeneity and not rescue of suboptimal provirus expression by Tat *in trans.* Additionally, we report a previously unappreciated phenomenon of enhanced double infection within primary T_CM_ cells and suggest that these long-lived cells may serve as an archive that drive ongoing viral recombination events in vivo.

HIV-1 has been transmitted from non-human primates to humans on at least four separate occasions, giving rise to HIV-1 groups M, N, O, and P [1–4]. HIV-1 group M, which accounts for the vast majority of infections worldwide, is believed to have been transmitted from chimpanzees to humans in the early 20th century [5, 6]. SIVcpz, the simian immunodeficiency virus infecting chimpanzees and the precursor of HIV-1, is the result of recombination between primate immunodeficiency viruses from red-capped mangabeys (SIVrcm) and greater spot-nosed monkeys (SIVgsn) [7]. Following transmission to humans, HIV-1 group M subsequently diversified into phylogenetically distinct subtypes labeled A1, A2, B, C, D, F1, F2, G, H, J and K. In addition, more than 70 circulating recombinant forms (CRFs) have been identified ([8] and the Los Alamos National Laboratory HIV sequence database (http://www.hiv.lanl.gov/content/sequence/HIV/CRFs/CRFs.html)).

The role of recombination in the HIV-1 epidemic is not purely historical but rather continues to contribute to the remarkable genetic heterogeneity of viral sequences both within infected individuals as well as on a population level. In infected individuals, recombination helps drive the rapid evolution of a diverse and complex viral population from a small number of initial founder viruses [9] and has adverse implications for drug resistance and immune escape [10, 11]. On an epidemiological scale, the genetic diversity of HIV-1 variants presents a significant challenge to vaccine design [11].

Molecularly, recombination occurs as the viral reverse transcriptase switches between two co-packaged genomic RNAs. The diversity engendered by recombination has been estimated to be on a similar frequency as the nucleotide substitution rate in patients, with an average of 1.4×10^−5^ recombinations per site per cycle [12]. Viruses produced from a cell infected by a single HIV variant have essentially identical viral genomes due to the low error rate of host RNA polymerase II. Therefore, while recombination can contribute to mutagenesis and may account for 15–20 % of all mutations occurring during reverse transcription [13], recombination can occur in viruses from a singly infected cell but does not lead to substantial reshuffling of viral genomes. A different situation arises in cells that are infected by two distinct HIV viruses: here, viruses co-package potentially diverse RNA genomes and recombination during subsequent infection of host cells can produce chimeric viruses. Thus, a pre-requisite for recombination events leading to significant reshuffling of viral genomes is the infection of host cells with two or more genetically distinct viruses, or ‘double infection’ of host cells [14].

In this study we investigated double infection of primary CD4+ T cells using reporter viruses expressing two distinct fluorescent proteins, EGFP and mCherry. We confirm previous reports that double infection of host cells occurs more frequently than would be expected by chance alone [15–17]. This non-random enhancement of double infection has been proposed to be the result of cellular heterogeneity [17] or rescue of suboptimal proviral expression by Tat *in trans* [15]; however, this latter mechanism did not account for enhanced double infection rates in primary CD4+ T cells using our combination reporter virus system. Instead, we observed that the majority of CD4+ T cells in the peripheral blood are refractory to HIV infection regardless of titer and that single and double infection are restricted to a small population of cells. Interestingly, double infection occurred preferentially in central memory CD4+ T cells compared to naïve CD4+ T cells. This phenomenon, which was independent of enhanced SAMHD1-mediated restriction of HIV in naïve CD4+ T cells, suggests that long-lived central memory CD4+ T cells are preferential targets for double infection and may represent an archived population that is an ongoing source of virions carrying distinct RNA genomes and driving recombination in vivo.

## Methods

### Cells

This study was conducted according to the principles specified in the Declaration of Helsinki and under local ethical guidelines (Case Western Reserve University Institutional Review Board (IRB)). Normal donor samples were de-identified and obtained from leukapheresis from ALLCELLS, LLC. All donors were negative for HBV, HCV, and HIV. A 10 year-old male with the homozygous mutation c.1411-2A > G in the *samhd1* gene, as reported previously [18], was recruited from the DDC Clinic (Middlefield, OH) with informed consent. The patient was on low-dose steroid (5 mg prednisone) treating the underlying condition while the samples were obtained. CD4+ T cells were isolated by adding additional autologous red blood cells to leukapheresis samples and RosetteSep CD4+ T cell enrichment kit antibodies (STEMCELL Technologies) prior to ficoll gradient separation. Cells were cryopreserved and treated with benzonase upon thawing, prior to infection. CD4+ T cells from the patient with confirmed *samhd1* mutations were purified by negative selection from freshly isolated PBMCs using a CD4+ T cell enrichment kit (STEMCELL Technologies).

### Production of viruses

Combination reporter viruses were produced as previously described [19]. Briefly, HEK 293T cells were seeded in 10 cm dishes and cultured overnight prior to transfection with 10.0 μg pNL4-3-deltaE-EGFP (obtained through the NIH AIDS Research and Reference Reagent Program, Division of AIDS, NIAID, NIH: pNL4-3-deltaE-EGFP (Cat #11100) from Drs. Haili Zhang, Yan Zhou, and Robert Siliciano), 7.5 μg bla-Vpr plasmid (obtained from Dr. Robert Doms), and 6.0 μg of HIV Env (CXCR4-tropic HIV Envs: JOTO.TA1.2247 [20] (obtained from Drs. Beatrice Hahn and George Shaw), IIIB/LAI, and TYBE; CCR5-tropic HIV Envs: REJO.D12.1972 [21](Obtained from Drs. Hahn and Shaw), ADA, and JR-CSF) using calcium phosphate methods [22]. Using the same protocol, viruses used in co-infection experiments were made using 10.0 μg of either pNL4-3-deltaE-EGFP or 10.0 μg pNL4-3- deltaE-mCherry (produced by replacing the *egfp* gene in the pNL4-3-deltaE-EGFP plasmid with *mCherry*) and 6.0 μg of CXCR4-tropic HIV Env JOTO.TA1.2247. Virus was harvested 72 h after transfection, filtered, and concentrated by ultracentrifugation through a 20 % sucrose cushion according to published protocols [23]. A p24 ELISA (Cell Biolabs, Inc.) was done with all viral stocks to determine viral concentrations. Empirical titers were also determined by infecting primary CD4+ T cells and evaluating viral fusion (for bla-Vpr containing viruses) or reporter gene positivity (for viruses lacking the bla-Vpr plasmid).

### Infection experiments

For titration experiments, infections were set up in parallel plates. One plate was used to measure viral fusion 24 h post-infection, while the other plate was used to measure productive infection 72 h post-infection. A range of 1 to 2048 ng of p24-equivalent viral stock was added to 5 × 10^5^ cells per well of a 96-well V-bottom plate. Cells were plated in RPMI media with 10 % FBS and 1 % L-glutamine, spinoculated at 1200 × g at 25 °C for 2 h and incubated at 37 °C for 1 h. No exogenous stimuli, such as mitogens, CD3/CD28, or IL-2, were added. The productive infection plate was transferred to corresponding wells of a 96-well flat bottom plate and incubated at 37 °C for staining at 72 h post-infection. Cells in the fusion plate were washed with CO_2_-independent media (Gibco) and incubated with CCF2-AM dye (Invitrogen) for 1 h at room temperature according to manufacturer instructions. Cells were then washed and incubated in CO_2_-independent media containing 5 % human serum (Gemini Bio-Products) and 2.5 mM probenecid (Sigma Aldrich) overnight at room temperature for staining 24 h post-infection. For co-infection experiments, 125 or 250 ng p24-equivalents of viral stocks were added to 10^6^ primary unstimulated CD4+ T cells in wells of a 96-well V-bottom plate. Cells were spinoculated at 1200 × g at 25 °C for 2 h, incubated at 37 °C for 1 h, and subsequently transferred to corresponding wells of a 96-well flat bottom plate. Cells were incubated for 72 h at 37 °C prior to staining for flow cytometry. Infection conditions were assayed in duplicate. For deoxynucleoside treatment of cells, 2’-deoxyadenosine, 2’deoxyguanosine, thymidine, and 2’deoxycytidine hydrochloride were purchased from Sigma Aldrich. Deoxynucleosides were dissolved in RPMI containing 10 % FBS and 1 % penicillin/streptomycin. Cells were incubated in 1 mM dNs for 1 h at 37 °C prior to spinoculation as described above.

### Flow cytometry

For titration experiments, cells were stained with anti-human CCR7 IgM (BD Biosciences) and fixable yellow dead cell stain (Invitrogen) at 37 °C for 30 min, washed, and incubated with anti-human CD3 BV650 (Biolegend), CD4 APC (eBioscience), CD45RO ECD (Beckman Coulter), and anti-IgM PE (Invitrogen) at 4 °C for 30 min. For coinfection experiments, cells were stained with anti-human CCR7 IgM (Becton Dickinson) and fixable yellow dead cell stain (Invitrogen) at 37 °C for 30 min, washed, and incubated with anti-human CD3 BV650 (Biolegend), CD4 APC (eBioscience), CD45RA APC-Cy7 (Biolegend), and anti-IgM PE (Invitrogen) at 4 °C for 30 min. Upon completion of antibody incubations, cells were washed with PBS and resuspended in 1 % paraformaldehyde in PBS/BSA prior to analysis using a BD LSRII flow cytometer. At least 50,000 events were collected per sample. FlowJo version 9.6 (Tree Star, Inc.) was used for analysis.

### Statistics

Unless otherwise specified**,** data in figures represent mean values and standard error of the mean. All *p* values were corrected for multiple comparisons where appropriate. Statistical analyses were performed using the paired *T*-test using GraphPad Prism v6.0e.

## Results

We have recently developed an HIV-1 combination reporter virus system that monitors both fusion and LTR-driven EGFP expression–a surrogate of production infection–in the viral life cycle and provides insight into the efficiency and outcome of infection [19]. Briefly, combination reporter viruses are produced by co-transfecting a packaging cell line with an NL4-3 core containing an *egfp* gene in the place of *env* gp120, a plasmid encoding a *β-lactamase–vpr* (*bla-vpr*) fusion gene, and a plasmid expressing an envelope gene, typically R5- or X4-tropic HIV *env*. The resultant virions contain bla-Vpr protein, which cleaves the β-lactamase substrate CCF2-AM in cells following fusion, eliminating a fluorescent resonance energy transfer (FRET) linkage and altering the fluorescent characteristics of the cell. If the virions successfully complete reverse transcription, nuclear import, integration, and viral long terminal repeat (LTR)-driven transcription, EGFP is produced in the cells and can be detected by flow cytometry.

**Fig. 1:**
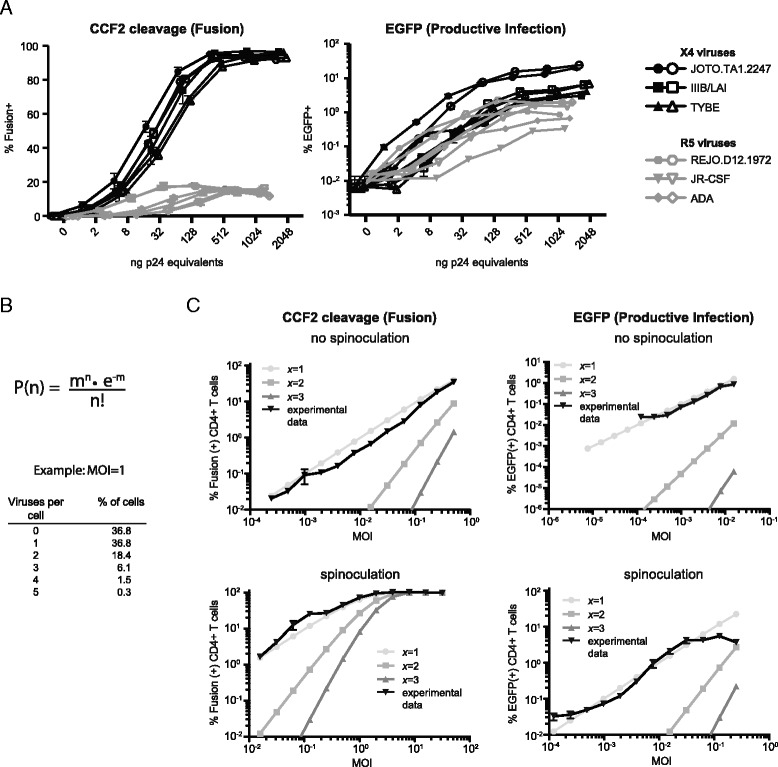
**a** Titration of combination reporter viruses bearing the CXCR4-tropic HIV Envs JOTO.TA1.2248, IIIB/LAI, or TYBE or the CCR5-tropic Envs REJO.D12.1972, JRCSF, or ADA. Flow cytometry was used to measure the percentage of fusion(+) CD4+ T cells by detection of CCF2-AM dye cleavage and EGFP(+) cells by the combination of EGFP expression and Nef-mediated CD4 downregulation. Closed and open symbols represent data from separate normal donors. **b** Equation for Poisson distribution to predict the percentage of cells (*P(n)*) infected with *n* viruses. *m* is the multiplicity of infection (MOI) and *e* is Euler’s number. The example shows the expected frequency of cells infected by 0, 1, 2, 3, 4, or 5 cells at a MOI of 1. **c** Representative experiment (1 of 3) showing the percentage of unstimulated CD4+ T cells undergoing fusion (*left*) or expressing EGFP (right) following infection with 1–2048 ng p24 equivalents of combination reporter viruses pseudotyped with the primary X4-tropic isolate JOTO.TA1.2247. These experimental data are compared with data predicted by Poisson distribution if a threshold (*x*) number of 1, 2, or 3 viruses is required for signal detection. Thresholds greater than *x* = 3 have increasingly steep slopes that do not match the experimental data. Multiplicity of infection (MOI) values were calculated by determining the MOI that best fit the experimental data using the Poisson distribution formula. MOI varied depending on the readout (fusion or EGFP signal) and whether the cells were spinoculated or not despite a constant number of cells and identical viral titrations. (Note: titrations <16 ng p24 equivalents were not included for EGFP in the absence of spinoculation because these concentrations did not result in signals above the background fluorescence of 0.02–0.03 % in the EGFP channel)

Using this system, we performed titrations of combination reporter viruses bearing CCR5- or CXCR4-tropic HIV envelopes (Fig. 1a). The three combination reporter viruses bearing CCR5-tropic viruses showed similar maximal levels of fusion (~15 % of peripheral blood CD4+ T cells), consistent with our previous findings [19]. In contrast, combination reporter viruses bearing CXCR4-tropic HIV Envs fused with 90–95 % of CD4+ T cells. EGFP expression levels were also higher for CXCR4-tropic HIV compared with CCR5-tropic HIV (7.61 ± 2.69 % EGFP+ v. 1.21 ± 0.28 %).

To gain further insight into the stoichiometry of HIV-1 infection using this system, we performed additional experiments monitoring both viral fusion and LTR-driven EGFP expression over a wider input range of reporter virus with the goal of determining the number of viruses required to produce a detectable cleaved CCF2-AM or EGFP signal in infected cells. A CXCR4-tropic HIV Env was chosen for these studies to facilitate study of HIV infection of both naïve and memory cells and because the extended range of fusion and EGFP values facilitated accurate modeling analysis. The probability of a cell being infected by *n* viruses [*P(n)*] can be determined using the Poisson distribution equation $P(n)=\frac{m^n\bullet {e}^{-m}}{n!}$ where *m* is the multiplicity of infection (MOI), and *e* is Euler’s number (Fig. 1b). Although multiple infection events in a given cell cannot be distinguished using this assay, cells can be identified as either uninfected or infected. If a single virus is sufficient to provide a cleaved CCF2 or EGFP signal, only cells with no virus [*P(0)*] will be detected as uninfected while cells with one or more viruses [*P(1) + P(2) + P(3)…*] will be detected as infected cells. In contrast, if a threshold (*x*) number of viruses are required to generate a signal, all cells infected with less than the threshold [*P(0) + … + P(x-1)*] will be counted as ‘uninfected’ while those at the threshold or above [*P(x) + P(x + 1) + …*] will be detected as ‘infected’. The practical consequence of this observation is that as viral MOI is lowered, ‘infected’ cells decrease at characteristic rates depending how many viruses are required for a signal to be generated. We performed titration experiments using 2-fold increases of HIV X4-tropic combination reporter viruses from 1 to 2048 ng p24 equivalents. These data were compared to decay curves calculated by Poisson distribution at thresholds of 1, 2, and 3 viruses (*x* = 1, 2, and 3, respectively) required to generate fusion or LTR-driven EGFP signals (Fig. 1c). The experimental data tracked with the decay curves with a threshold of 1, indicating that a single virus was sufficient to detect CCF2 cleavage and EGFP signals. Two deviations from the theoretical data were noted for EGFP signal. The deviation at low viral input is due to background fluorescence in the EGFP channel of approximately 0.02–0.03 % of CD4+ T cells and is observed even in uninfected samples. For this reason, viral inputs <16 ng p24 equivalents are not included in the EGFP “no spinoculation” experiments because only background signal is detectable. The second deviation at high viral input in the presence of spinoculation is not a technical limitation but rather reflects the finding that only a proportion of unstimulated peripheral blood CD4+ T cells are capable of being productively infected regardless of viral titer, as will be discussed further.

### Cells infected by more than one virus occur at higher than expected frequencies

**Fig. 2:**
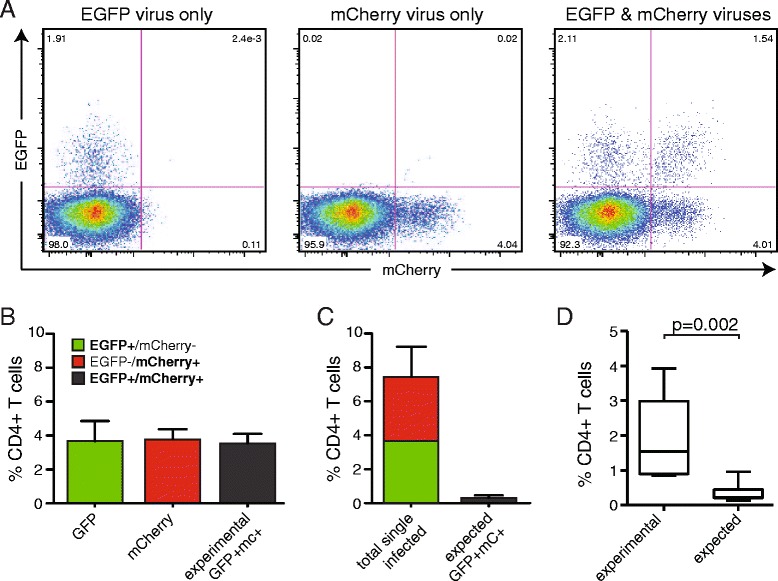
**a** Cells infected with viruses expressing EGFP, mCherry, or both can be distinguished by flow cytometry. Representative data (1 of 9) of unstimulated primary CD4+ T cells infected with NL4-3-ΔE-EGFP and/or NL4-3-ΔE-mCherry reporter viruses pseudotyped with the primary X4-tropic isolate JOTO.TA1.2247. **b** Frequencies of EGFP+/mCherry-, EGFP-/mCherry+, and EGFP+/mCherry + cells 72 h after infection of unstimulated CD4+ T cells with EGFP and mCherry viruses, normalized by MOI. **c** For a given percentage of single positive cells (*P(1),* EGFP+mCherry- and EGFP-mCherry+), the expected frequency of double positive cells (*P(2)*) can be predicted by Poisson distribution. **d** Comparison of actual frequencies of double positive cells measured experimentally and frequences expected by Poisson distribution. The data are from experiments on 9 different healthy controls

Several studies have previously reported that cells infected by more than one virus occur at a higher than expected frequency in both primary cells and cell lines [15–17]. To investigate this phenomenon, we infected purified, unstimulated CD4+ T cells with viruses encoding either *egfp* or *mCherry* reporter genes (Fig. 2a), normalized by MOI. Cells expressing only EGFP or mCherry were counted as singly infected cells and cells expressing both EGFP and mCherry were counted as doubly infected (representative experiment shown in Fig. 2b). Using the observed frequency of singly infected cells, we used the Poisson distribution to determine the expected percentage of cells with two or more viruses (Fig. 2c). As mentioned previously, we are unable to distinguish cells infected with one or multiple copies of the same reporter gene, so these likely reflect slight overestimates of the actual frequency of singly infected cells and slight underestimates of doubly infected cells. Despite this bias, and in agreement with previous studies, we observed a statistically greater percentage of doubly infected cells than predicted by Poisson distribution, indicating nonrandom enhancement of double infection (experimental: 1.88 ± 0.38 % double infected; expected: 0.35 ± 0.08 %, *p* = 0.002, Fig. 2d).

### Higher than expected frequencies of double infection are not due to suboptimal provirus expression rescued by trans effects of Tat

**Fig. 3:**
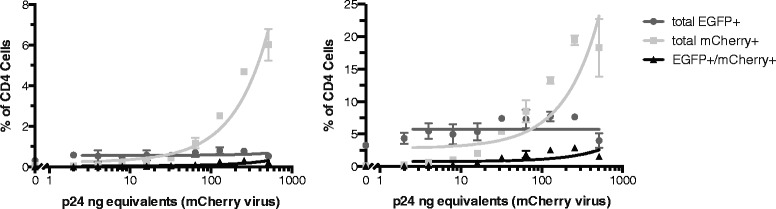
Unstimulated CD4+ T cells were infected with a constant input of 40 ng of X4-pseudotyped EGFP virus and increasing concentrations of X4-pseudotyped mCherry virus over a range from 0 to 512 ng. The slope of the total EGFP+ cells (EGFP+/mCherry- and EGFP+/mCherry+) was not significantly different than 0 (*p* > 0.5 for both healthy controls). The slope of total mCherry + cells and doubly infected (EGFP + mCherry+) cells were both significantly different than 0 (total mCherry + cells: *p* < 0.0001 for both controls; doubly infected cells: *p* = 0.0004 and *p* = 0.015, respectively). Representative data from 2 (of 4) experiments

Efficient expression of viral mRNAs from the HIV LTR promoter requires the expression of viral Tat in *cis* to recruit the elongation factor P-TEFb to the viral transactivating region (TAR) element of nascent mRNAs, phosphorylate the C terminal domain of RNA Pol II, and relieve the block to RNA elongation (recently reviewed by Mbonye and Karn [24]). This Tat-mediated regulation of the LTR promoter activity is a positive feedback loop: Tat protein enhances viral mRNA production, leading to higher Tat protein levels that in turn further enhance promoter activity. In contrast, if Tat protein levels are insufficient to recruit P-TEFb, HIV mRNAs stall at ~59 bp in length and Tat levels are further diminished, a phenomenon frequently referred to as suboptimal provirus expression or direct silencing. One potential explanation for the higher than expected frequencies of doubly infected cells is that suboptimal provirus expression could be rescued by Tat in *trans*, i.e. by Tat produced by a second integrated provirus. Indeed, Tat provided by a second virus can rescue reporter gene expression from a Tat-defective virus in HeLa-CD4 cells [15]. To evaluate whether suboptimal provirus expression was also occurring in primary CD4+ T cells, we performed experiments where cells from two healthy controls were infected with a constant amount of EGFP-expressing virus (40 ng p24 equivalents) and mCherry-expressing viruses were added at increasing concentrations from 0 to 512 ng p24 equivalents. We reasoned that if suboptimal provirus expression was occurring and could be rescued by Tat in *trans, * then the percentage of EGFP+ cells should increase with higher levels of mCherry viruses as they activated silenced LTR promoters of *egfp* proviruses. However, while the slope of mCherry expression was significantly different than zero (*p* < 0.0001 for both healthy controls), the slope of EGFP expression was not (*p* > 0.50 for both, Fig. 3), indicating that mCherry viruses had no effect on the percentage of cells expressing EGFP. These data demonstrate that Tat insufficiency and rescue in *trans* is not responsible for the enhanced double infection of primary CD4+ T cells.

### Only a fraction of unstimulated CD4+ T cells in the peripheral blood can become infected with HIV

**Fig. 4:**
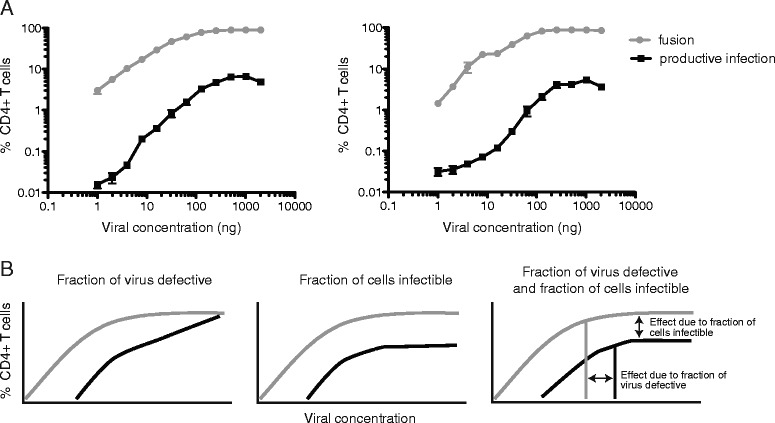
Only a fraction of primary CD4+ T cells are susceptible to HIV infection regardless of viral titer. **a** Representative fusion and productive infection (EGFP) data from 2 (of 4) healthy controls is shown. While viral fusion reaches a plateau at ~85–95 % of CD4+ T cells undergoing fusion—consistent with expression of CXCR4—EGFP expression reaches a plateau at far lower levels (less than 15 % for all donors tested). **b** Modeling of expected percentage of cells undergoing fusion or productive infection as a function of viral concentration. Different curves are expected if a fraction of virus is defective, a fraction of cells are infectible, or both

An alternative explanation to the higher than expected frequencies of double infection is that only a fraction of cells are susceptible to HIV infection. If this hypothesis is correct, single and double infection would be expected to occur relatively frequently in the susceptible population but rarely or not at all in non-susceptible cells. We empirically tested this hypothesis in primary CD4+ T cells through further titration experiments including extremely high inputs of HIV reporter viruses. Cells from two normal donors were infected with combination reporter viruses at inputs between 1 and 2048 ng p24 equivalents. Fusion of HIV with CD4+ T cells increased with viral input until concentrations of 256 ng and above, at which point a plateau was reached with 85–90 % of CD4+ T cells fusing with HIV (Fig. 4a). Consistent with our previous findings [19], the percentage of cells undergoing fusion was ~10–20–fold higher than the percentage of cells expressing LTR-driven EGFP. There are several potential explanations for this phenomenon, including (a) a proportion of viruses that are defective for producing EGFP, (b) cells that can fuse with HIV but are refractory to productive infection, or (c) both (Fig. 4b). Importantly, if defective viral particles are solely responsible for the difference between fusion and EGFP signals, the percentage of cells expressing EGFP should continue to increase over the entire range of the input virus. In contrast, if only a fraction of CD4+ T cells are susceptible to HIV, the percentage of cells expressing EGFP would be expected to plateau once the susceptible population has been completely infected. The experimental data from both donors clearly demonstrate a plateau of EGFP+ CD4+ T cells, indicating that only a fraction of primary CD4+ T cells are susceptible to HIV regardless of viral concentration. Although CCR5 and CXCR4-tropic viruses differ markedly in the percentage of cells that are susceptible to fusion (Fig. 1a), the percentage of cells that progress to EGFP expression in both cases is only ~8 % of the total number of fusion(+) cells (R5-tropic viruses: 8.99 ± 2.35 %, X4-tropic viruses, 8.13 ± 2.88 %, *p* > 0.5), suggesting that regardless of the coreceptor used for entry or the percentage of lymphocytes than the virus can enter, only a fraction of unstimulated peripheral blood CD4+ cells can be productively infected. It should be noted that the ‘defective viruses’ in Fig. 4b refers to viruses that are incapable of producing EGFP in the combination reporter virus assay. In vivo, ‘defective interfering particles’ or DIPs with genomic deletions have been reported to replicate at the expense of wild-type virus; however these inhibitory effects occur at the level of genome incorporation—downstream of LTR-driven EGFP expression—and do not explain the plateauing of EGFP at high levels of input virus observed here.

### Double infection is disfavored in naïve CD4+ T cells and occurs preferentially in the central memory CD4+ T cells

**Fig. 5:**
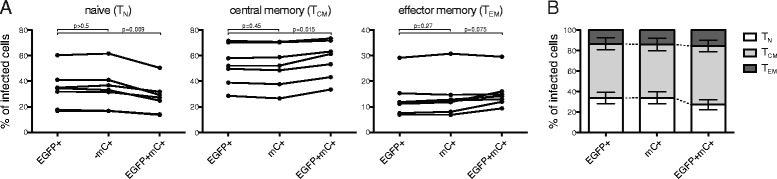
Central memory and effector memory cells constitute a larger percentage of doubly infected cells compared to singly infected cells. **a** The frequencies of naïve (T_N_), central memory (T_CM_) and effector memory (T_EM_) cells within EGFP+/mCherry- (EGFP+), EGFP-/mCherry + (mC+), and EGFP + mCherry + (EGFP + mC+) populations. Data are independent experiments in 7 healthy controls. **b** Contribution of T_N_, T_CM_, and T_EM_ to singly and doubly infected CD4+ T cells

To determine whether double infection occurs stochastically across all CD4+ T cell subsets or is favored in certain subsets compared to others, we compared the contribution of naïve (T_N_, CCR7 + CD45RO-), central memory (T_CM_, CCR7 + CD45RO+), and effector memory (T_EM_, CCR7-CD45RO+) subsets to singly (EGFP+ or mCherry+) and doubly-infected (EGFP + mCherry+) cells in 7 donors infected with 1:1 ratios of EGFP and mCherry reporter viruses. Terminal effector (T_TE_, CCR7-CD45RO-) CD4+ T cells were present at very low frequencies and were not included in the analysis. No significant differences were observed in subset distribution of cells infected by EGFP or mCherry viruses only (T_N_: 33.88 ± 5.58 % EGFP+ v. 33.96 ± 5.81 % mCherry+, *p* > 0.5; T_CM_: 52.76 ± 5.93 % v. 52.14 ± 6.17 %, *p* = 0.45; T_EM_: 13.36 ± 2.84 v. 13.91 ± 2.99 %, *p* = 0.27, Fig. 5a). In contrast, we observed that T_N_ CD4+ T cells comprised a significantly lower percentage of doubly infected cells compared to singly infected cells (33.88 ± 5.58 % EGFP+ cells v. 27.30 ± 4.71 % EGFP + mCherry + cells, *p* = 0.009), whereas T_CM_ were over-represented among doubly infected cells (52.76 ± 5.93 % EGFP+ cells v. 57.14 ± 5.56 % EGFP + mCherry + cells, *p* = 0.015). T_EM_ cells were enriched among double positive cells but did not reach statistical significance after corrections for multiple comparisons (13.36 ± 2.84 v. 15.56 ± 2.47 %, *p* = 0.075). These data demonstrate that although double infection occurs less frequently than single infection in all subsets, double infection occurs less frequently in naïve cells compared to memory cells, resulting in a preferential enrichment of memory cells–particularly T_CM_–among doubly-infected cells (Fig. 5b).

### SAMHD1 restricts viral replication in CD4+ T cells but is not responsible for preferential double infection of memory subsets

**Fig. 6:**
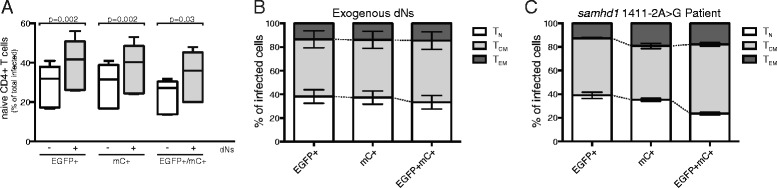
SAMHD1-mediated restriction of HIV infection in naïve CD4+ T cells does not account for the differential distribution of naïve (T_N_), central memory (T_CM_), and effector memory (T_EM_) subsets among singly (EGFP+ or mC+) and doubly infected (EGFP+ mC+) cells. **a** Bypassing SAMHD1-mediated regulation of intracellular nucleotide pools via addition of exogenous dNs increased the representation of naïve CD4+ cells in singly and doubly infected cell populations, indicating that SAMHD1 restricts HIV-1 infection to a greater extent in naïve cells compared to memory cells. **b** Addition of exogenous dNs did not eliminate the under-representation of naïve T cells among doubly infected cells compared to singly infected cells (*p* = 0.002). **c** A patient with sequence-confirmed mutations in *samhd1* also demonstrated increased contribution of T_CM_ cells and decreased contribution of T_N_ cells to the doubly infected cell population

The greater representation of memory cells–and particularly central memory cells–among doubly-infected cells could be the result of either (1) memory cells being more susceptible to double infection or (2) naïve cells being refractory to double infection. We have previously found that naïve CD4+ T cells are refractory to HIV infection compared with memory cells, with a smaller percentage progressing to LTR-driven EGFP expression following fusion [19, 25]. This was in part due to SAMHD1-mediated restriction of HIV infection in naïve cells, as knockdown of SAMHD1 via Vpx-containing viral like particles (VLPs) resulted in an ~20-fold increase in EGFP expression in the T_N_ subset compared to only 5–8–fold increases in T_CM_ and T_EM_ subsets [25]. We reasoned that if SAMHD1 restricted entry of a single virus in T_N_ cells to a greater extent than in T_CM_ and T_EM_ cells, the likelihood of two viruses successfully completing reverse transcription, integration, and gene expression would be multiplicatively more rare in T_N_ cells and could drive the observed changes in subset distribution. To test this hypothesis, we infected cells with 1:1 ratios of EGFP and mCherry reporter viruses in the presence or absence of 1 mM dNs, which bypasses the SAMHD1-mediated reduction of intracellular nucleotide pools and facilitates viral reverse transcription [26, 27]. Treatment with exogenous dNs increased the total infection levels and the percentage of single and double infected cells of the T_N_ phenotype (*p* = 0.002 and *p* = 0.03, respectively, Fig. 6a), consistent with our previous finding that SAMHD1 was a more potent restrictor of HIV infection in T_N_ cells than in T_CM_ or T_EM_ cells. However, even in the presence of exogenous dNs, T_N_ cells were under-represented among doubly infected cells compared to singly infected cells (*p* = 0.006, Fig. 6b). Since SAMHD1 has recently been described to have anti-HIV activity that is independent of its action in regulating intracellular nucleotide pools [28], we also tested the susceptibility of cells from a patient with the homozygous mutation c.1411-2A > G in the *samhd1* gene to infection with HIV. This mutation occurs in the splice acceptor site of intron 12 of SAMHD1, leading to the skipping of exon 13, an in-frame 31 amino acid deletion, and the nearly complete absence of SAMHD1 protein by Western blot analysis [18]. Consistent with the results of the exogenous dN experiments, cells from the T_N_ subset were represented at reduced frequencies among doubly infected cells compared with singly infected cells in this patient (Fig. 6c). Together, these data indicate that while SAMHD1 restricts HIV infection in CD4+ T cells—particular the T_N_ subset—it is not responsible for the preferential inhibition of double infection in naïve T cells that results in the skewed distribution of doubly-infected cells towards memory subsets, particularly T_CM_ cells.

## Discussion

In this study, we investigated double infection of primary CD4+ T cells using combination reporter viruses that measure viral fusion and LTR-driven EGFP or mCherry expression. In agreement with previous studies, double infection of CD4+ T cells occurred more frequently than expected. Several explanations for enhanced double infection have been proposed, including (i) a fraction of uninfectable target cells [17], (ii) variation in cellular susceptibility to HIV [17], and (iii) suboptimal proviral expression rescued by a second provirus via Tat expression in *trans* [15]. Whereas the experiments performed in this study demonstrate the presence of uninfectable target cells and the differential susceptibility of CD4+ T cell subsets to HIV, no evidence for activation of silent proviruses in *trans* was observed. Intriguingly, double infection did not occur stochastically across all CD4+ T cells maturation subsets but rather occurred preferentially in central memory (T_CM_) cells. As SAMHD1 restricts viral infection to a greater extent in naïve CD4+ T cells compared with memory cells [25], we speculated that it could further reduce double infection of T_N_ cells and drive the observed subset differences between single and double infection. However, preferential double infection of T_CM_ cells was maintained even after bypassing SAMHD1-mediated restriction of intracellular nucleotide pools with exogenous nucleotides or in a patient with the homozygous mutation c.1411-2A > G in the *samhd1* gene, suggesting other, currently unknown, factors influence preferential double infection of T_CM_ cells.

From an experimental perspective, the titration experiments performed with the combination reporter viruses not only demonstrate that a single reporter virus is sufficient for fusion and EGFP signals, but also provide insight into the use of multiplicity of infection (MOI) in normalizing for viral input and into the effect of spinoculation on viral infection. MOI, as originally defined by Max Delbrück in studies of T4 bacteriophage in the 1930s, referred to the ratio of infectious virions to host cells using a plaque counting assay. As CD4+ T cells grow in suspension, it is not possible to ‘count’ the number of replication competent viral foci in culture. The most accurate method for calculating HIV titers is a limiting dilution-infection assay and the determination of the viral concentration required to infect 50 % of the wells, or TCID_50_. As this assay is laborious, surrogate methods for determining titers have been developed that rely upon detection of infected cells by reporter gene expression, integrated DNA, or even total DNA and using these assays to calculate MOI. If all viruses were infectious and all cells universally susceptible to infection, these surrogates could be used interchangeably and would provide accurate assessment of viral titers. However, for HIV this is clearly not the case and the above surrogates provide increasingly inaccurate measures of productive infection. Intuitively, the earlier in the viral life cycle the surrogate measurement is taken, the higher the proportion of cells that will appear infected and the less accurately these assays will predict true, replication-competent viral titers. For instance, our titration data demonstrate that measuring viral infectivity using an early life-cycle indicator (CCF2 dye cleavage) results in ~120-fold higher MOIs than a late life-cycle indicator (LTR-driven EGFP expression). In part, these discrepancies are likely a result of defective viral particles: based on p24 concentrations and an estimate of roughly 5000 Gag molecules per virus [29], the number of potential particles is ~1,300-fold higher than the fusion-competent particles and ~160,000-fold higher than the particles capable of giving rise to integration and LTR-driven EGFP expression. These values are consistent with ratios of noninfectious or inert virus particles per infectious units (P/IU) of 10^2^–10^7^ for HIV (recently reviewed by Klasse [30]). In addition, these discrepancies between fusion and EGFP measurements likely reflect heterogeneity in cellular susceptibility to infection: more cells are susceptible to fusion with HIV than are permissive to integration and LTR-driven EGFP expression and, presumably, still fewer cells are fully susceptible to HIV replication. Surrogates for measuring HIV titers are inherently imperfect, but our data indicate that assays measuring late HIV life cycle stages will be more accurate than those measuring early stages. These considerations may be useful for the planning and interpretation of experiments relying on normalizing viruses by MOI.

Spinoculation, the experimental technique of centrifuging cells in the presence of virus, has been demonstrated to dramatically increase infection rates of target cells [31]. Underscoring the profound effect of spinoculation on HIV infection, MOIs for fusion and EGFP were ~60–fold and 15–fold higher in the spinoculation conditions, respectively. In other words, spinoculation had an effect on viral infectivity equivalent to adding between 15- and 60-fold more virus to the cells. Exactly how spinoculation acts to promote viral infection remains unclear, although recent reports suggest that it may disrupt the cortical actin barrier and facilitate viral entry into cells [32]. Our findings suggest that the primary effect of spinoculation occurs upstream of the readout of the fusion assay; however, the precise requirements for bla-Vpr–mediated CCF2 cleavage remain unknown. For instance, migration of the virus through the cortical actin barrier, dissociation of the p17 matrix shell of the virion, and perhaps additional intracellular steps may be required to allow bla-Vpr to interact with the CCF2 substrate, making ‘fusion assay’ a slight misnomer. Additionally, we observed that spinoculation improved the ‘fusion’ assay readout to a greater extent than EGFP expression—60-fold and 15-fold compared to no spinoculation, respectively—suggesting that spinoculation may actually reduce the efficiency of viral life cycle events between the stages of CCF2 dye conversion and LTR-driven EGFP expression. Further investigation into how spinoculation improves infectivity of primary cells will be of considerable value in understanding barriers to HIV infection and improving *ex vivo* transduction techniques.

From a biological perspective, the results from our study suggest that the observed enhanced double infection rates in primary CD4+ T cells are primarily a result of only a fraction of cells that can be infected by HIV-1 regardless of titer. Within this susceptible population, single and double infection could be following the expected Poisson distribution but the large proportion of cells resistant to the virus gives the appearance of enhanced double infection in the total CD4+ T cell population. Additionally, we also observed that naïve CD4+ T cells were more refractory to HIV infection following fusion than memory subsets, in agreement with our previous findings [19]. Presciently, in their initial report [17], Dang and colleagues proposed that non-random enhancement of double infection could be a consequence of cells resistant to HIV infection or due to heterogeneity of primary CD4+ T cell populations, hypotheses that have become testable and confirmed here via the development of the combination reporter virus assay that monitors multiple stages of the viral life cycle.

In contrast, we did not observe evidence for rescue of proviral insufficiency by the production of Tat in *trans* by a second provirus, as has been reported by Brégnard and colleagues [15]. In agreement with Brégnard, we and others have previously demonstrated that HIV can become directly silenced following infection events as evidenced by enhancement of frequency of EGFP(+) cells following stimulation with anti-CD3/CD28, SAHA, or TNF-α. However, we found no evidence of enhanced EGFP expression in cells infected with a constant level of EGFP viruses and increasing concentrations of mCherry viruses, suggesting that infection with a second provirus did not reactivate silenced promoters via production of Tat in *trans.* However, our experimental system differs from that used by Brégnard in key ways that likely account for these discrepant findings. First, in several experiments Brégnard and colleagues provided Tat in *trans* via transfection rather than infection, which would likely result in different levels of Tat protein expression than native infection. Second, the most definitive experiments demonstrating rescue by Tat *in trans* were performed using HeLa or HPB-ALL cells, which are both immortalized cell lines that may differ from primary CD4+ T cells in significant ways such as the availability of transcription factors for the LTR promoter. Indeed, we predict that the cells undergoing immediate silencing following infection have cellular conditions unfavorable to viral replication—such as insufficient nuclear NF-κB to drive transcription—and that a second provirus entering into these cells would encounter the same unfavorable environment and also undergo silencing following integration.

The experimental system used in this manuscript describes near-simultaneous infection of cells by two distinct viruses, or co-infection. Another potential route of double infection in vivo is sequential infection where a cell becomes infected with a virus and is subsequently infected with a second virus at a later time. Which of these routes predominates in patients is not clear; however, HIV has mechanisms to prevent sequential infection at the cellular level, including Nef-mediated CD4+ down-regulation. Indeed, in our assay, unstimulated cells infected with a virus encoding *nef* have undetectable levels of CD4 by 3 days after infection [19] and become resistant to infection with a second virus. In activated cells, where reverse transcription and integration occur more efficiently, CD4 is likely down-regulated even more rapidly. We believe that in the context of an active HIV infection, particularly in lymphoid tissues such as lymph node or spleen, the level of viral replication in vivo could conceivably lead to cells being exposed to multiple viruses in a short time frame similar to the experimental conditions used here. Studies of splenocytes from HIV-infected patients have indeed demonstrated the presence of cells infected with multiple viruses [33, 34], suggesting that cells were exposed to multiple viruses in this cell-rich environment either simultaneously or in rapid succession, prior to down-regulation of CD4.

Finally, the finding that double infection did not occur stochastically across all subsets but rather was favored in central memory (T_CM_) cells further confirms the heterogeneity of CD4+ T cells with respect to HIV infection. As T_CM_ cells are among the longest-lived subsets of CD4+ T cells and are a primary contributor to the latent reservoir in patients treated with antiretroviral therapy [35], their preferential double infection implies that these cells may represent an archived population of cells with multiple proviruses that can drive ongoing recombination in vivo, with significant implications for the reshuffling of viral genomes during periods of treatment interruption or intermittent viremia.
